# A Comparative Analysis of the Venom Gland Transcriptomes of the Fishing Spiders *Dolomedes mizhoanus* and *Dolomedes sulfurous*

**DOI:** 10.1371/journal.pone.0139908

**Published:** 2015-10-07

**Authors:** Xunxun Xu, Hengyun Wang, Fang Zhang, Zhaotun Hu, Songping Liang, Zhonghua Liu

**Affiliations:** College of Life Sciences, Hunan Normal University, Changsha, 410081, Hunan, China; Weizmann Institute of Science, ISRAEL

## Abstract

*Dolomedes sulfurous* and *Dolomedes mizhoanus* are predaceous arthropods catching and feeding on small fish. They live in the same area and have similar habits. Their venoms exhibit some similarities and differences in biochemical and electrophysiological properties. In the present work, we first performed a transcriptomic analysis by constructing the venom gland cDNA library of *D*. *sulfurous* and 127 novel putative toxin sequences were consequently identified, which were classified into eight families. This venom gland transcriptome was then compared with that of *D*. *mizhoanus*, which revealed that the putative toxins from both spider venoms might have originated from the same gene ancestors although novel toxins were evolved independently in the two spiders. The putative toxins from both spiders contain 6–12 cysteine residues forming seven cysteine patterns. As revealed by blast search, the two venoms are rich in neurotoxins targeting ion channels with pharmacological and therapeutic significance. This study provides insight into the venoms of two closely related species of spider, which will be of use for future investigations into the structure and function of their toxins.

## Introduction

The spiders known to catch and feed on small fish and insects are particularly large, semi-aquatic spiders (fishing spiders) [1, 2]. The genus *Dolomedes* are the most dominant group of fishing spiders, which are predators with a leg span of 6–9 cm and a weight of 0.5–2 g [3]. There are more than 90 species in *Dolomedes*. *Dolomedes sulfurous* and *Dolomedes mizhoanus*, mainly distributed in the south of China, live in the same geographical region and have the similar diet habits. The venoms from the two spiders have been investigated in our previous researches [4, 5]. They contain hundreds of peptides with different molecular weight distribution as revealed by off-line RP-HPLC/MALDI-TOF-MS analysis; they potentially inhibit voltage-activated Na^+^, K^+^, and Ca^2+^ channels in rat dorsal root ganglion neurons; and the intrathoracic injection of the two venoms at high concentrations caused severe neurotoxic symptom and death of zebrafish, respectively. It was also found that *D*. *mizhoanus* venom has lower inhibitory potency compared with *D*. *sulfurous* venom. These data indicate that the peptide toxins from the two venoms have some similarities and differences [4, 5]. A comparative analysis of their peptide toxins should provide insight into the diversity of the toxins from the two closely related species of spider.

Due to the limited crude venoms collected from the two spiders, it is hard to carry on the investigation of individual toxins through venom purification. Alternatively, venom gland transcriptomic analysis may be employed to determine the sequences of cDNA and therefore the encoded sequences of peptide toxins are derived [6]. In light of that, a comparative analysis of the venom gland transcriptomes of the two spiders was conducted in the present study. The transcriptomic information of the venom gland cDNA library of *D*. *mizhoanus* has been illustrated in our previous study [7]. Therefore, we constructed the venom gland cDNA library of *D*. *sulfurous* in this study. As a result, 267 high-quality expressed sequence tags (ESTs) were generated and 127 novel putative toxin sequences were identified.

## Materials and Methods

### cDNA library construction

A directional full-length *D*. *sulfurous* venom gland cDNA library was constructed by the same method as that to the venom gland cDNA library of *D*. *mizhoanus* [7]. Four days after being milked via electrical stimulation, venom glands from ten female adult spiders were obtained and homogenized in liquid nitrogen. The spiders were collected by ourselves near the Xiushui River in Guilin in Guangxi province and maintained in our laboratory. The spiders are not endangered species and therefore no permission was required for the areas where the spiders were collected. Total RNA was extracted with RNAiso plus (TaKaRa biotechnology (Dalian) Co. Ltd.). 1.0 μg total RNA was used for library construction. The full-length cDNA library synthesis was completed according to the instructions for the Creator^TM^ SMART^TM^ cDNA Library Construction Kit (Clontech Laboratories, Inc). The inserted cDNAs in the individual colonies were amplified by PCR using general M13 forward and reverse primers. The PCR products were subjected to electrophoresis on a 1% agarose gel, which determined the size of each product. Selected clones with cDNA length being >400 base pairs were sequenced by using an ABI 3730 automatic DNA sequencer according to the manufacturer’s instructions (Shanghai Sangon Biological Engineering Technology and Service Co., Ltd., Shanghai, China).

### Expression sequence tags sequencing and bioinformatic analysis

After removing the PolyA tail, short sequences were discarded and high-quality sequences were assembled into clusters using SeqMan Pro module of DNASTAR Lasergene software suite. cDNA sequences (contigs and singletons) were used to search against public databases (nr/NCBI, Swissprot +TREMBL/EMBL) by using the BlastX program with the e-value cutoff set to <10^−5^ to identify similar sequences and putative functions of the new ESTs [8,9]. Signal peptides were predicted with the SignalP 3.0 program [http//www.cbs.dtu.dk/services/SignalP/] [10]. The phylogenetic analysis of putative toxins was conducted by the MEGA 5 software using the neighbor-joining method and bootstrap values estimated from 1000 replicates [11]. Multiple sequence alignment was performed by the ClustalW2 program on the basis of amino acid sequence similarity [12, 13].

## Results

### cDNA library and EST analysis

**Fig 1:**
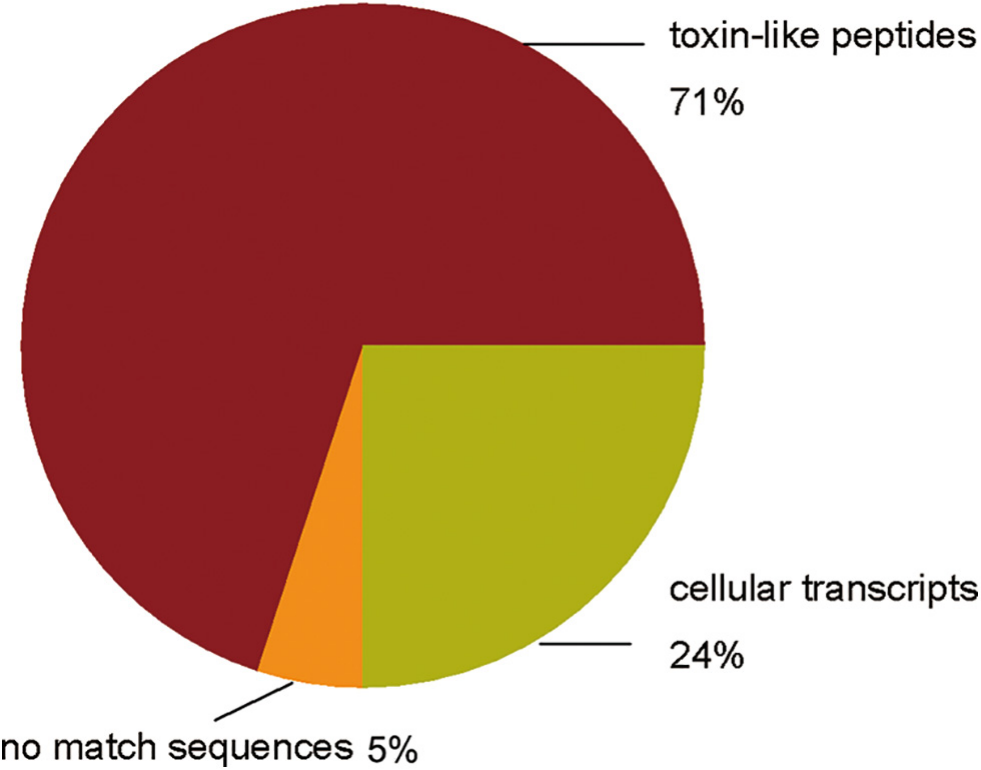
Relative proportion of each transcript category from *D*. *sulfurous* venom gland cDNA library.

**Fig 2:**
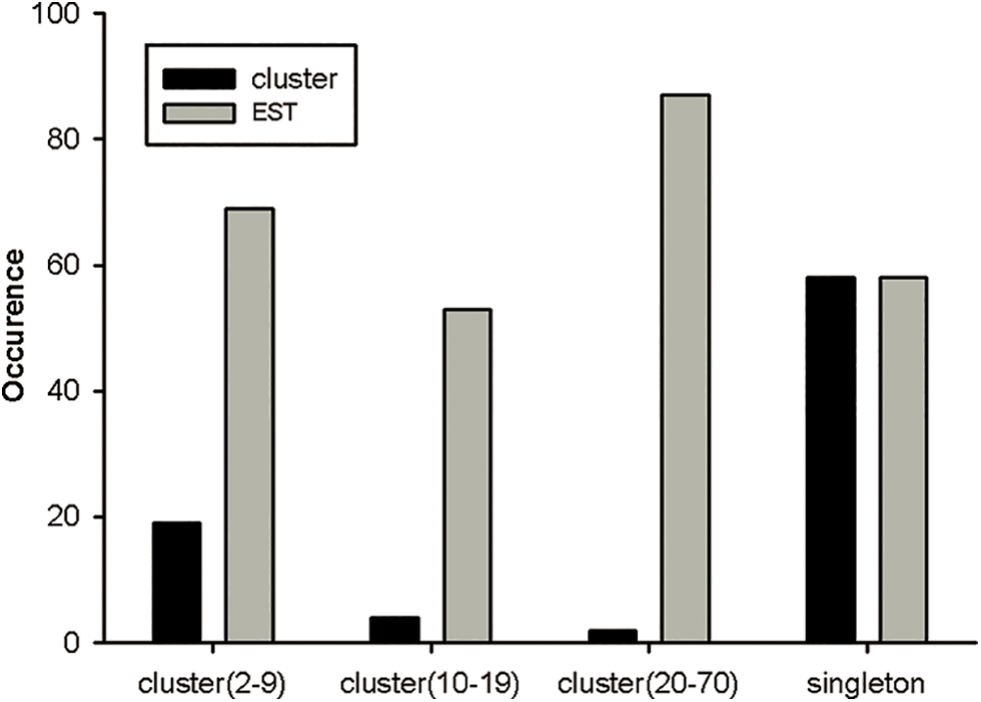
Prevalence distribution of the cluster size. The initial 267 ESTs were grouped into 25 contigs and 58 singletons.

The directional full-length cDNA library was generated from the venom glands of *D*. *sulfurous*. The average length of the cDNA in the library was about 500 bp, ranging from 0.3 to 1.0 kb. After removing vector, short and poor-quality sequences, 267 high-quality ESTs were randomly generated from the cDNA library. Of the 267 ESTs, 71% (190 of 267 ESTs) might encode putative toxin precursors, 24% (65 of 267 ESTs) were similar to cellular transcripts, and 5% (12 of 267 ESTs) had no significant similarity to any known sequences (Fig 1). The average readable sequence length was approximately 599 bp. 204 non-redundancy sequences from the total 267 ESTs encoded 127 novel putative toxin precursors (GenBank accession numbers, KP777610-KP777736), 65 cellular body proteins (GenBank accession numbers, KP777737-KP777801) and 12 unknown proteins which had very low sequence similarity with any known proteins, respectively. Of the 127 putative toxin precursors, 90 non-redundant mature peptide sequences were obtained. The 267 ESTs assembling resulted in 83 clusters, including 25 contigs and 58 singletons. The abundance distribution of all ESTs was cataloged as follows (Fig 2): (1) Two clusters contained 20–70 ESTs, respectively, which represented 2.4% of the total clusters (2 of 83 clusters) and 32.6% of the total ESTs (87 of 267 ESTs). All of them were predicted to encode toxins. (2) Four clusters included 10–19 ESTs, respectively, which represented 4.8% of the total clusters (4 of 83 clusters) and 19.8% of the total ESTs (53 of 267 ESTs). They might encode toxins and cellular proteins. (3) 19 clusters included 2–9 ESTs, respectively, represented 22.9% of the total clusters (19 of 83 clusters) and 26% of the total ESTs (69 of 267 ESTs). (4) There were 58 singletons representing 21.6% of ESTs (58 of 267 ESTs) and 69.9% of the total clusters (58 of 83 clusters), which were unique ESTs and the occurrence rate of these clusters was only once in the library.

In our previous study, the venom of *D*. *sulfurous* was analyzed by RP-HPLC (S1 Fig) and the molecular weights of some peptide toxins were determined by MALDI-TOF MS. On the other hand, the molecular weights of cDNA-deduced peptide toxins could be calculated according to putative mature toxin sequences. By comparing these two kinds of molecular weights, the closest matching would be found and therefore the amino acid sequences corresponding to the determined molecular weights and eluted peaks in RP-HPLC could be obtained. As shown in S1 Table, six mature peptide sequences are finally determined by closest matching. This also indicated that the real presence of the corresponding transcripts in the venom gland. It should be noted that the number of toxin sequences determined by this strategy was much less than that of putative toxin sequences derived from cDNA sequences. This might be caused by the reasons as follows: (1) some peptide toxins are present in very low amounts, which were not able to be detected by MALDI-TOF MS analysis; (2) some peptide toxins might have some post-translation modification, which causes the real molecular weights different from the calculated ones. In our further study, *de novo* sequencing of HPLC fractions by MS will be used to determine more peptide sequences based on our transcriptomic data.

### Classification of toxin-like precursors

**Fig 3:**
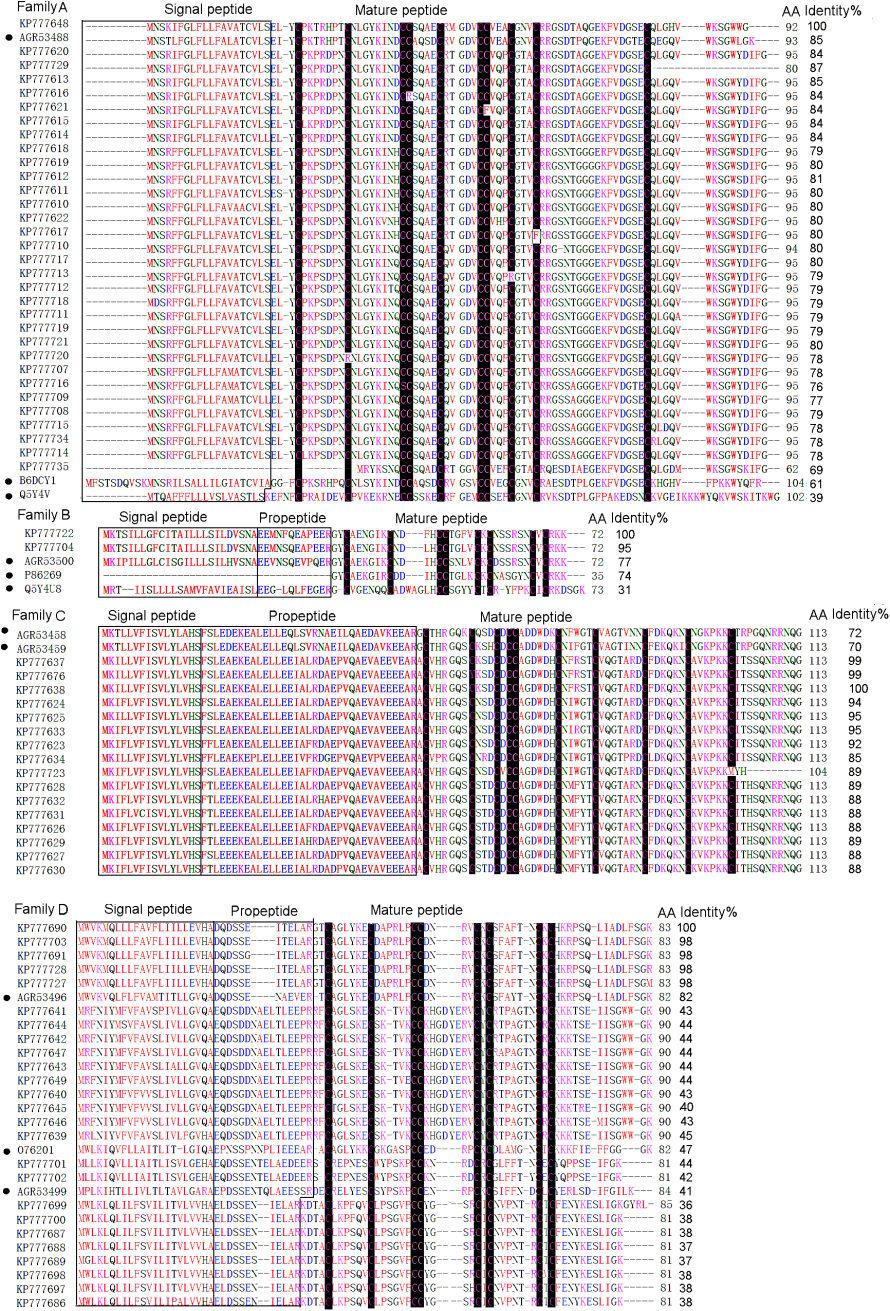
Multiple sequence alignment of putative toxin precursors from the cDNA library of *D*. *sulfurous*. Cysteine residues are shaded in black. The signal peptides and propeptides are shown in boxes. The UniProtKB/Swiss-Prot accession numbers of the known toxins are as follows, LSTX-Q4, B6DCY1; AgorTX_A5, Q5Y4V; Purotoxin–1, P86269; Mu-2Aaga_12, Q5Y4U8; PNTx3-2, O76201. The GeneBank accession numbers of the known toxins are as follows, DMTX–60, AGR53488; DMTX–41, AGR53500; DMTX–154, AGR53458; DMTX–207, AGR53459; DMTX–3, AGR53496; DMTX–104, AGR53449.

**Fig 4:**
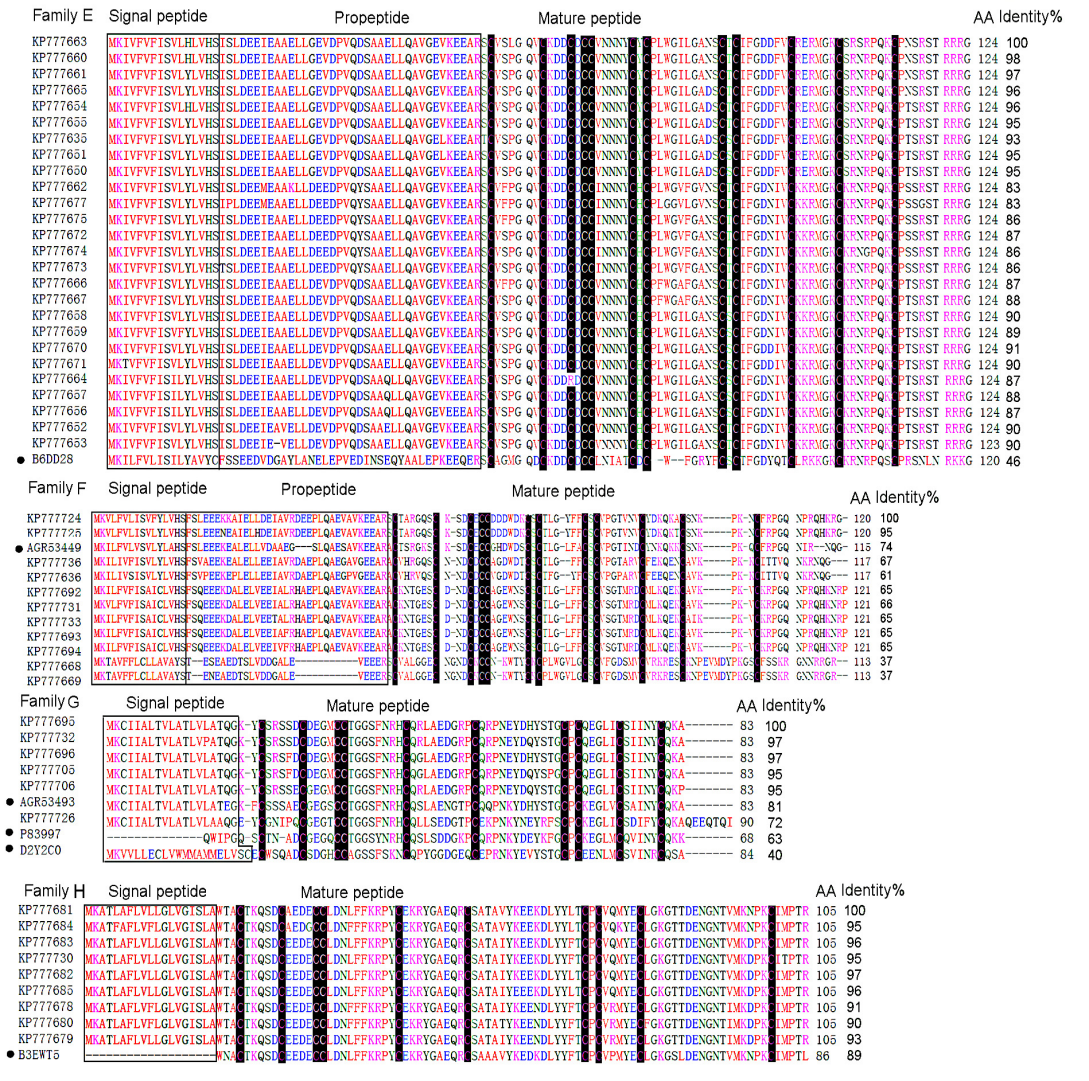
Multiple sequence alignment of putative toxin precursors from the cDNA library of *D*. *sulfurous*. Cysteine residues are shaded in black. The signal peptides and propeptides are shown in boxes. The UniProtKB/Swiss-Prot accession numbers of the known toxins are as follows, LSTX-L10, B6DD28; CSTX–20, B3EWT5; U19-CNTX-Pn1a, P83997; HNTX-XIV, D2Y2C0. The GeneBank accession numbers of the known toxins are as follows, DMTX–61, AGR53499; DMTX–12, AGR53493.

All the putative toxin precursors from this cDNA library were classified into eight families (A-H) according to phylogenetic analysis and cysteine patterns (Figs 3 and 4).

#### Family A

The family A was composed of 32 putative toxin precursors (Fig 3), which was the most abundant cluster in this library and showed extremely high identity (85%) with DMTX–60 (GeneBank No., AGR53488) identified from *D*. *mizhoanus* [7]. It consisted of signal peptide and mature peptide and was absent of propeptide region. Some of the precursors contained a single residue “G” at the C-terminals, indicating C-terminal amidation during post-translational process. The mature sequences of this family were composed of 60–75 amino acid residues, including 10 cysteine residues and the same cysteine pattern (–Cx_7_-Cx_8_-CCx_4_-Cx_5_-CCx_3_-Cx_3_-Cx_17_-C-) with two pairs of CC fragments, which were extremely rare in other spider toxins identified so far. This cysteine pattern was as same as that of U20-lycotoxin-Ls1d(LSTX-Q4)(UniProtKB/Swiss-Prot No., B6DCY1) from the spider *L*. *singoriensis* and U7-agatoxin-Ao1a(AgorTX_A5)(UniProtKB/Swiss-Prot No., Q5Y4V) from the spider *A*. *orientalis* [14, 15]. It was interesting that the 10 cysteine residues were unevenly distributed along the amino acid sequences of this family, of which nine were located in the N-terminal and only one was in the C-terminal, indicating that the C-terminal structure was flexible with less disulfide bond constraints.

#### Family B

The family B contained two putative toxin precursors which had the same signal peptides and a single site mutation (F/S) in the propeptide region (Fig 3). They differed by only two amino acid residues in the mature peptides, implying that they were natural mutants. The family exhibited high sequence identity (77%) with DMTX–41(GeneBank No, AGR53500) from *D*. *mizhoanus* [7]. The predicted mature peptides had 35 amino acid residues with 8 cysteines in the pattern of “-Cx_6_C-CC-CxC-CxC-”. The mature peptides showed 74% and 31% sequence identities with purotoxin–1 (UniProtKB/Swiss-Prot No.,P86269) from the spider *Geolycosa sp*. and U3-AGTX-Ao1k (μ-2Aaga_12) (UniProtKB/Swiss-Prot No., Q5Y4U8) from the spider *Agelena orientalis*, respectively [15,16]. Purotoxin–1 selectively inhibits P2X3 receptor and has an analgesic effect in rats. μ-2Aaga_12 is an insecticidal neurotoxin that modulates the insect voltage-gated Na^+^ channels in a unique manner. The data suggest that the two putative toxins KP777704 and KP777722 in this family might possess the similar activities to purotoxin–1 or μ-2Aaga_12.

#### Family C

The family C contained 16 putative toxin precursors which showed 72% and 70% sequence identity to DMTX–154 (GeneBank No., AGR53458) and DMTX–207 (GeneBank No., AGR53459) from *D*.*mizhoanus* [7] (Fig 3). The predicted mature peptides had 53–62 amino acid residues, including 10 cysteine residues in the pattern of “-Cx_6_-Cx_3_-Cx-CCx_6_-Cx_5_-Cx_7_-Cx_6_-Cx_6_-C-”, expect KP777723 in which the last cysteine residue was replaced by methionine.

#### Family D

The family D contained 25 putative toxin precursors whose sequences were similar to DMTX–3 (GeneBank No., AGR53496) (82%) from *D*. *mizhoanus* [7] and ω-ctenitoxin-Pn1a (PNTx3-2) (UniProtKB/Swiss-Prot No., O76201) (47%) from *Phoneutria nigriventer*, an antagonist of L-type Ca^2+^ channels [17] (Fig 3). The predicted mature sequences were composed of 43–51 amino acid residues, of which eight cysteines formed the pattern of “Cx_6_-Cx_6_-CCx_4/8-_CxC-x_6/7_-CxC-”. The mature peptides had a particularly large number of basic residues corresponding to PI values in 8–9 range. The consensus residues “GK” at the C-terminals of the mature peptides were trimmed through post-translational amidation modification. The sequence similarity between this family and PNTx3-2 indicated that they might possess the potential of Ca^2+^ channel inhibitory activity.

#### Family E

There were 26 putative toxin precursors in this family which was the second abundant cluster in this library (Fig 4). The mature sequences had 70 amino acid residues and the primary structure was similar to U13-lycotoxin-Ls1b (LSTX-L10) (UniProtKB/Swiss-Prot No., B6DD28) from the spider *L*. *singoriensis* (46%) [14]. They contained 12 cysteine residues in the pattern of “-Cx_6_-Cx_3_-Cx-CC-x_5_-CxC-x_10_-CxC-x_7_-Cx_6_-Cx_7_-Cx_n_-”. It was noted that there was a single site mutation (C/R) in DSTX30 (GeneBank No., KP77664). This mutation led to the odd number of cysteine residues, which resulting in at least a pair of intrachain disulfide bond not to be formed.

#### Family F

There were 12 putative toxin precursors in this family (Fig 4). The predicted mature sequences were composed of 66–75 amino acid residues .12 cysteine residues formed the pattern of “-Cx_6_-Cx_3/4_-Cx-CC-x_5/6_-CxC-x_6/7_-CxC-x_7_-Cx_6_-Cx_6/12_-Cx_n_-”, which is similar to that of Family E, expect different residue numbers between two closed cysteine residues. The mature sequences showed 74% sequence identity to DMTX–104 (GeneBank No., AGR53449) from *D*.*mizhoanus* [7].

#### Family G

Six putative toxin precursors belonged to this family (Fig 4). It was also lack of propeptide region. The mature peptides (64 amino acid residues) included 10 cysteine residues which formed a consensus cysteine pattern “-Cx_5_-Cx_4_-CC-x_8_-C-x_9_-C-x_12_- CxC-x_5_-C-x_5_-C-”. These peptides were highly similar to DMTX–12 (GeneBank No., AGR53493) (81%) from *D*. *mizhoanus* [7]. Blast search presented homologous toxins Hainantoxin-XIV (HNTX-XIV) (UniProtKB/Swiss-Prot No., D2Y2C0) (40%) from the spider *Haplopelma hainanum* [18] and U19-ctenitoxin-Pn1a (PNTx16C1) (UniProtKB/Swiss-Prot No., P83997) (63%) from *Phoneutria nigriventer* which was non-toxic to mice and insects.

#### Family H

The family H that was also lack of propeptide region contained nine putative toxin precursors (Fig 4). The mature sequences had 86 amino acid residues, in which 10 cysteine residues were in the arrangement of Cx_5_-Cx_4_-CC-x_10_-Cx_9_-Cx_16_-CxC-x_5_-Cx_18_-Cx_n_. Unlike putative toxins from other families, the mature sequences in this family were rich in acidic residues with the PI values in the 4–5 range. Another unique property of this family was that they had no similarity to the toxins from *D*. *mizhoanus*. They exhibited homology (89%) to CSTX–20 (UniProtKB/Swiss-Prot No., B3EWT5) from the spider *Cupiennius salei*. This spider was commonly called American wandering spider or hunting spider distributed in the tropical rainforest of South and Central America [19]. However, the function of this peptide remains unknown.

### Phylogenetic analysis of putative toxins from the two spider venoms

As indicated above, most putative toxin precursors from *D*. *sulfurous* share very high sequence similarity with those from *D*. *mizhoanus*. Therefore, all putative toxin precursors from the two spiders (127 from the former and 53 from the latter) were mixed and submitted to multiple sequence alignment and phylogenetic analyses. The multiple sequence alignment was performed by using ClustalW, indicating that all 180 precursors could be categorized into nine families (Family I-IX) (S2 Fig). Four families (Families I-III and VIII) contain precursors from the two spiders, showing these precursors of each family from the two spiders are very similar. On the other hand, three families (Families IV, V and VII) and two families (Families VI and IX) just included precursors from *D*. *mizhoanus* and *D*. *sulfurous*, respectively, indicating these precursors were different and had low sequence similarity.

**Fig 5:**
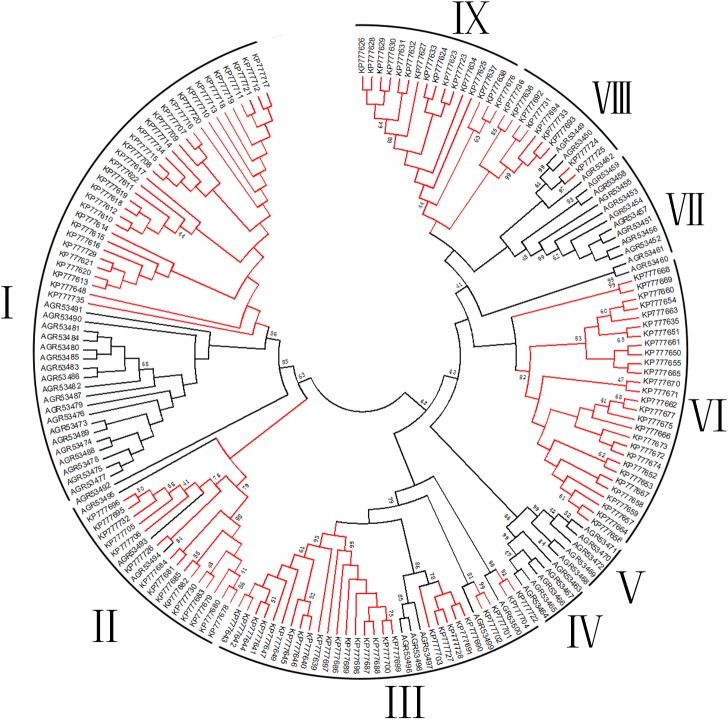
Phylogenetic tree of putative toxin precursors from *D*. *sulfurous* and *D*. *mizhoanus* venom glands. The phylogenetic analysis was conducted by using the Maximum Likelihood method of the MEGA 5 software package. Red and black branches represent putative toxin precusors from *D*. *sulfurous* and *D*. *mizhoanus*, respectively.

The phylogenetic tree of all precursors was drawn by using neighbor-joining method (S3 Fig) or maximum likelihood method (Fig 5). It should note that using the two methods exported similar phylogenetic trees. Consistent with the multiple sequence alignment data, nine families were indicated as represented by nine small clades in the phylogenetic trees (red and black branches represent precursors from *D*. *mizhoanus* and *D*. *sulfurous*, respectively). Generally speaking, all precursors formed two main clades, which suggested that peptide toxins from the two spiders might originate from same gene ancestors. The families I and II belonged to first main clade, while the remaining families III-IX formed the second one. In the families (Families I-III and VIII) containing precursors from the two spiders, the red and black branches mixed together, indicating these precursors were conserved during evolution process. On the other hand, in the families (Families IV-VII and V) formed by precursors from each spider species, respectively, the red or black branches were isolated to form independent small clades, indicating these precursors had different evolution directions in two spiders.

### Comparative analysis of cysteine patterns of putative toxins from the two spider venoms

**Fig 6:**
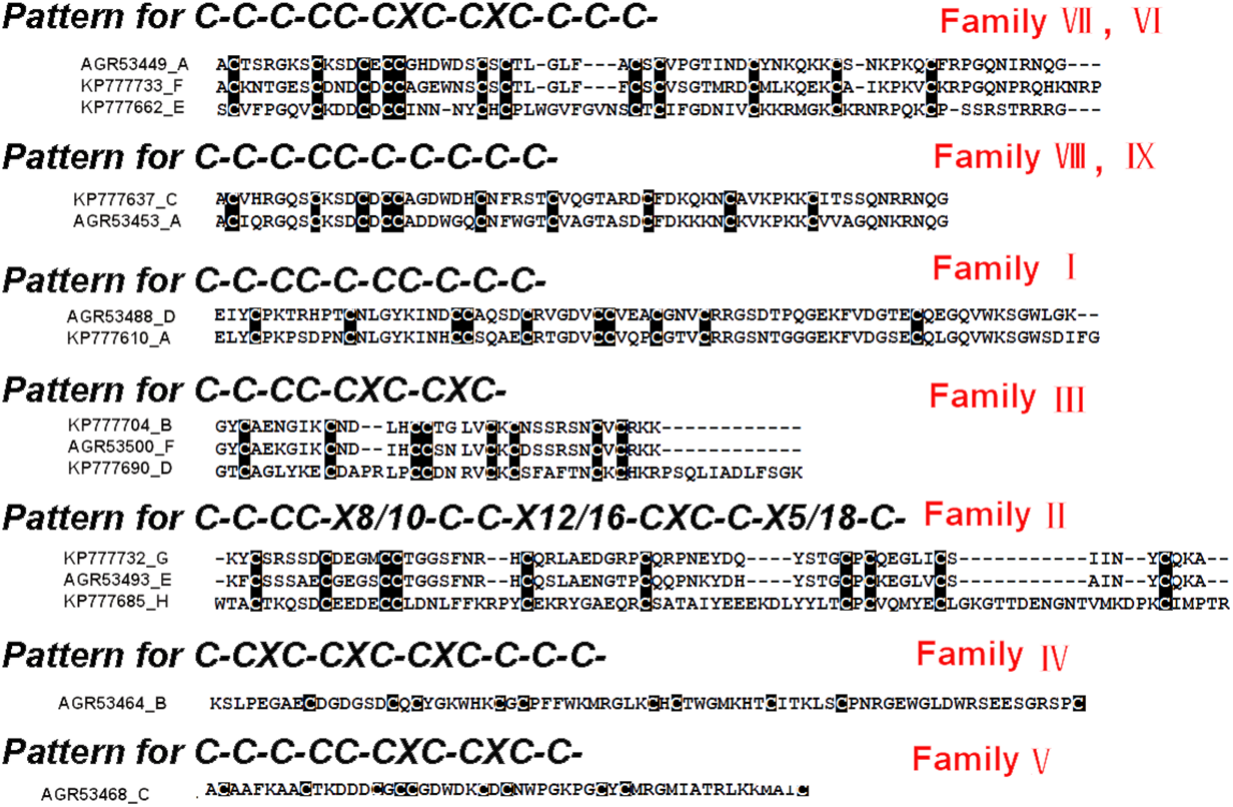
The total cysteine patterns of putative toxin peptides from *D*. *sulfurous* and *D*. *mizhoanus*. Within the cysteine patterns, the character “X” is any amino acid residues. The sequences are aligned using ClustalW2 and grouped based on the cysteine numbers and arrangement.

Peptide toxins from venomous animals are commonly rich in cysteine residues; which play an indispensable role in stabilizing their spatial structures. The patterns of cysteine residue distribution in toxin sequences (cysteine patterns) are closely correlated with disulfide linkages and space structures [20]. All the putative mature toxins from the two spider venoms contained 8–12 cysteine residues probably forming 4–6 disulfide bonds, which were different from toxins having traditional inhibitor cysteine knot (ICK) motif commonly formed by three or four disulfide bridges [20–23]. It was proposed that some of the putative toxins from the two venoms might adopt novel spatial structure scaffolds that are distinct from ICK motif. Seven different cysteine patterns were present in the toxin sequences from the two venoms. The seven cysteine patterns, the corresponding families and the representative toxin sequences from the two venoms are shown in Fig 6. Five patterns of them were shared by toxins from the two spiders, while the other two are specific to the toxins from *D*. *mizhoanus*. Generally speaking, the putative toxins containing the same cysteine pattern were distributed in the same families in the phylogenetic trees (Fig 5 and S3 Fig).

The cysteine pattern (C-C-CC-CXC-CXC-) was shared by putative toxins in the family III (Fig 6) from the two spiders. The special characteristic was the presence of the primary structure motif such Principal Structural Motif (PSM) and the Extra Structural Motif (ESM). PSM stipulates the existence of 6 amino acid residues between the first and second cysteine residue and there are no amino acids between the third and fourth cysteine residues. ESM means there is the presence of a pair of CXC fragments in C-terminal region. These widely appear in the ion channel blocking toxins and are suggested for the identification of the spider’s ion channel blocking toxins [15]. The presence of PSM and ESM in the putative toxins indicated above implied that they might function as blockers of ion channels, which are consistent with the blast analysis showing they share sequences similiarity with purotoxin–1 (P2X3 receptor antagonist) and PNTx3-2 (L-type Ca^2+^ channel blocker), respectively. Two patterns (C-CXC-CXC-CXC-C-C-C and C-C-C-CC-CXC-CXC-C) were only found in putative toxins in the families IV and V from *D*. *mizhoanus*, respectively. Accordingly, these putative toxins were distributed in two independent clades in the phylogenetic trees (Fig 5 and S3 Fig) without incorporation of the putative toxins from *D*. *sulfurous*.

## Discussion

Spider venoms are comprised of diverse neurotoxins, which are considered as powerful tools for the research of ion channels and have potential applications as novel pharmaceutical drugs [24–27]. In the present study, the transcriptomic approach and bioinformatic analysis were applied to investigate peptide toxins from *D*. *sulfurous* venom, which might provide clues for identifying individual toxins from this venom in the future study. Thereafter, a comparative analysis was conducted to clarify the similarities and differences of the peptide toxins from the venoms of *D*. *sulfurous* and *D*. *mizhoanus*, which might provide information for understanding the evolutionary relationship of the toxins from the two spiders that live in the same region and have similar habits.

From the available data, putative toxins from the venom of *D*. *sulfurous* are of great diversity (S2 Table). A total of 127 novel putative toxin precursors were deduced from 267 cDNA sequences, which can be divided into eight families. The putative toxins are expressed as precursors consisting of signal peptide, an intervening propeptide sequence and mature toxin sequence, except for families A, G and H toxins without propeptide sequences. The signal peptides consist of 16–25 amino acid residues and are relatively conserved. The propeptide sequences ranging from 12 to 38 amino acids contain nine different types of cleavage signals for the propeptide processing enzymes, most of which are the Processing Quadruplet Motif (PQM) just before the mature peptides. The mature toxins include variable length of amino acid residues (35–86); most mature toxins are long chain toxins with more than 50 amino acid residues, which are distinct from many tarantula toxins with 30–40 amino acid residues [23,28]. The mature sequences have 8–12 cysteine residues forming 4–6 disulfide bonds and display a variety of cysteine patterns which are also distinguished from most tarantula toxins identified so far [20,29,30]. Most of mature toxins possess C-terminal sequence motifs such as “G”, “GK”, “GR”, which are considered as amidation signals [31–33], indicating these putative toxins might be amidated in C-terminals during post-translational modifications.

To the point of toxin evolution, the putative toxins from the two spider venoms were close in the evolution distance and proposed to be derived from same gene ancestors as revealed by the phylogenetic analysis. Gene duplication and hypermutation, DNA insertion, deletion and replacement might generate novel sequences and structural motifs [34]. As a result, putative toxins with high sequence similarity are present in the two spider venom glands. On the other hand, the putative toxins from the two spiders might have distinct evolutionary directions, which consequently lead to produce very different putative toxins, respectively.

The cysteine pattern analysis highlights the structural diversities and specificity of the toxins from the two spiders. A total of seven cysteine patterns are observed among the putative toxins from the two spiders. Considering the important roles of cysteine patterns in stabilizing the spatial structures of peptide toxins, some of the putative toxins from the two spiders might adopt novel structure scaffolds, elucidation of which might expand our understanding of structural diversity of peptide toxins. In addition, of the seven cysteine patterns, five are shared by the putative toxins from the two spiders; the other two are only present in the putative toxins from *D*. *mizhoanus*, which further indicates that the spider *D*. *mizhoanus* might evolve some novel toxins during evolution.

Our previous studies indicated that the two venoms contain various neurotoxins targeting voltage-gated ion channels expressed in rat dorsal root ganglion neurons. In the present study, the activities of some putative toxins might be predicted according to their sequence identity to known toxins. Our data indicated that the venoms from the two spiders might be a useful source for identifying toxins with diverse functions. Most putative toxins might function as neurotoxins, for example, inhibitors of Ca^2+^ channels or P2X3 receptor. These channels and receptor are found to play important roles in some diseases, such as pain [35, 36], indicating that toxins targeting them might have therapeutic implications. In the future study, the cDNAs of these putative toxins will be synthesized and expressed in *E*. *coli* or yeast, and therefore their activities can be tested.

In summary, 127 novel putative toxin precursors were deduced from 267 cDNA sequences in *D*. *sulfurous* venom gland. The comparative analysis of the two spider venom gland transcriptomes indicated that their toxins might be derived from the common gene ancestors, but some novel toxins would be evolved independently in the two spiders during evolution. These toxins exhibited structural and functional diversity, which makes them a useful source for the identification of neurotoxins with pharmacological or therapeutic importance. The sequence determination and functional predication of these putative toxins might provide clues for the future studies.

## Supporting Information

S1 Fig RP-HPLC separation of the soluble venom from *D. sulfurous*. This was performed in an analytical C18 column equilibrated with solution A (distilled water in 0.1% TFA), using a gradient from 0 to 40% solution B (acetonitrile in 0.1% TFA) over 50 min, with a flow rate of 1 mL/min and absorbance at 215 nm. The fractions labelled with retention times show they might contain peptide toxins indicated in supplementary S1 Table.
(DOCX)

S2 Fig Multiple sequence alignment of putative toxin precursors from the cDNA library of *D*. *sulfurous and D*. *mizhoanus*. Cysteine residues are shaded in black. The signal peptides and propeptides are shown in boxes.
(DOCX)

S3 Fig Phylogenetic tree of putative toxin precursors from *D*. *sulfurous* and *D*. *mizhoanus*. The phylogenetic analysis was conducted by using the Neighbor-Joining method of the MEGA 5 software package. *D*. *sulfurous* and *D*. *mizhoanus* toxin precursors were marked with red and black lines, respectively.
(DOCX)

S1 Table Closest matches between molecular weights of peptide toxins determined by MALDI-TOF MS and calculated from putative mature toxin sequences of *D*. *sulfurous*
(DOCX)

S2 Table Molecular characters of the representative toxins in the families from the spider *D*. *sulfurous*. (DOCX)
