# Supplementary material for: A Comparative Analysis of the Venom Gland Transcriptomes of the Fishing Spiders Dolomedes mizhoanus and Dolomedes sulfurous
Source: PLoS One. 2015 Oct 7;10(10):e0139908. doi: 10.1371/journal.pone.0139908 (PMC4596850; doi:10.1371/journal.pone.0139908)
Supplement: S1 Fig — This was performed in an analytical C18 column equilibrated with solution A (distilled water in 0.1% TFA), using a gradient from 0 to 40% solution B (acetonitrile in 0.1% TFA) over 50 min, with a flow rate of 1 mL/min and absorbance at 215 nm. The fractions labelled with retention times show they might contain peptide toxins indicated in supplementary S1 Table. (DOCX) [file pone.0139908.s001.docx]

**
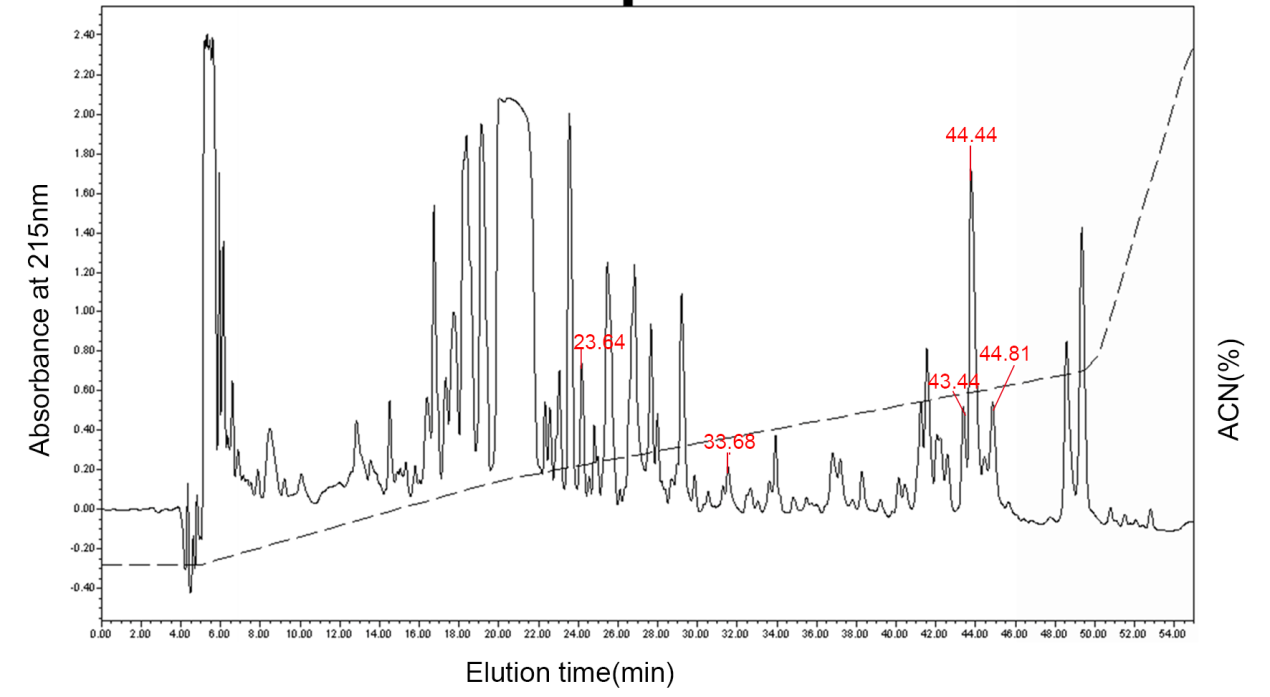
**

**Figure S1. RP-HPLC separation of the soluble venom from *D. sulfurous*.** This was performed in an analytical C18 column equilibrated with solution A (distilled water in 0.1% TFA), using a gradient from 0 to 40% solution B (acetonitrile in 0.1% TFA) over 50 min, with a flow rate of 1 mL/min and absorbance at 215 nm. The fractions labelled with retention times show they might contain peptide toxins indicated in Table S1.
