# Supplementary material for: A Comparative Analysis of the Venom Gland Transcriptomes of the Fishing Spiders Dolomedes mizhoanus and Dolomedes sulfurous
Source: PLoS One. 2015 Oct 7;10(10):e0139908. doi: 10.1371/journal.pone.0139908 (PMC4596850; doi:10.1371/journal.pone.0139908)
Supplement: S2 Fig — Cysteine residues are shaded in black. The signal peptides and propeptides are shown in boxes. (DOCX) [file pone.0139908.s002.docx]

**Figure S2**


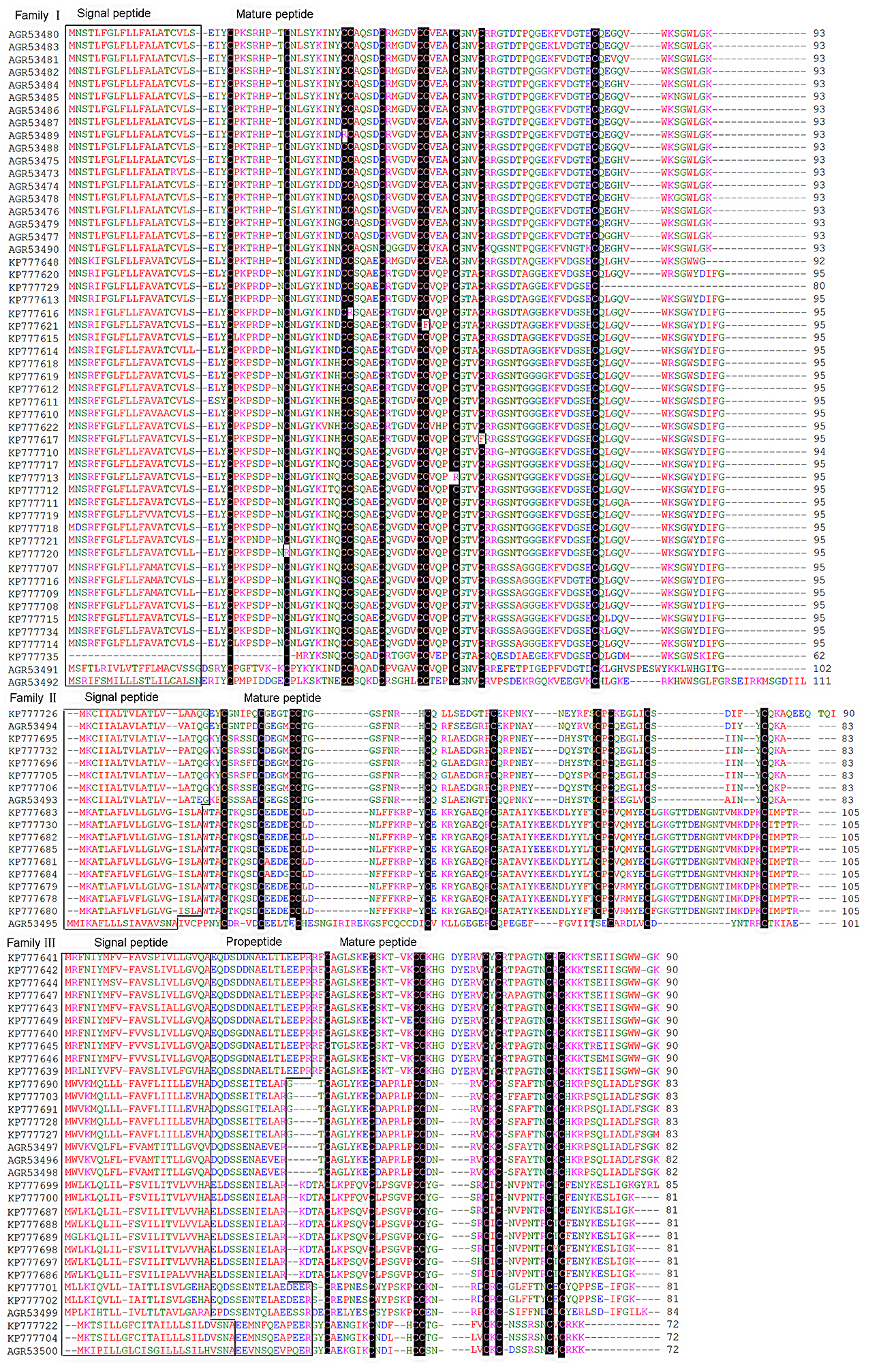


**Figure S2 continued**


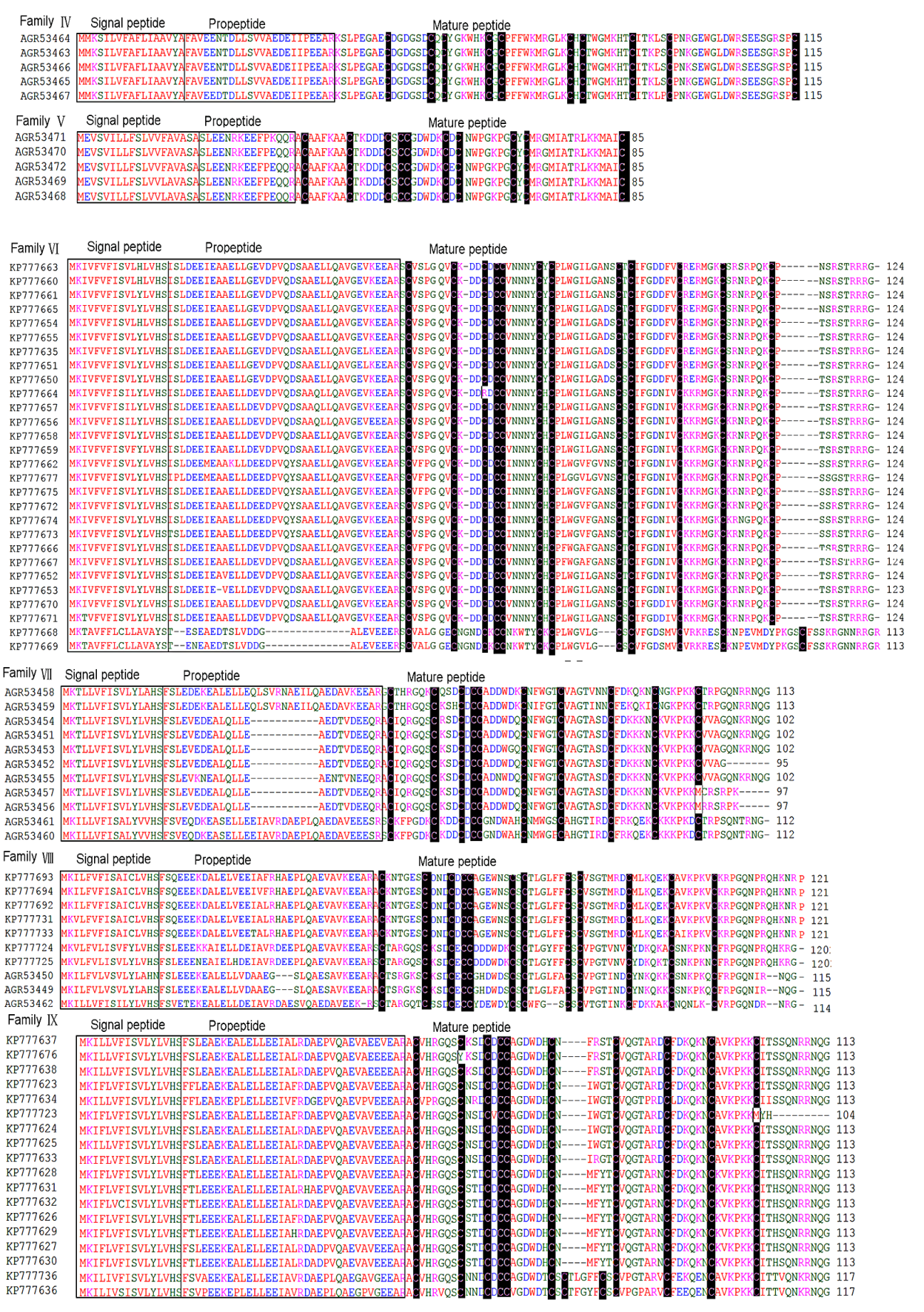


**Figure S2. Multiple sequence alignment of putative toxin precursors from the cDNA library of *D. sulfurous and D. mizhoanus*.** Cysteine residues are shaded in black. The signal peptides and propeptides are shown in boxes.
