# Supplementary material for: A Comparative Analysis of the Venom Gland Transcriptomes of the Fishing Spiders Dolomedes mizhoanus and Dolomedes sulfurous
Source: PLoS One. 2015 Oct 7;10(10):e0139908. doi: 10.1371/journal.pone.0139908 (PMC4596850; doi:10.1371/journal.pone.0139908)
Supplement: S3 Fig — The phylogenetic analysis was conducted by using the Neighbor-Joining method of the MEGA 5 software package. D. sulfurous and D. mizhoanus toxin precursors were marked with red and black lines, respectively. (DOCX) [file pone.0139908.s003.docx]

**Figure S3**


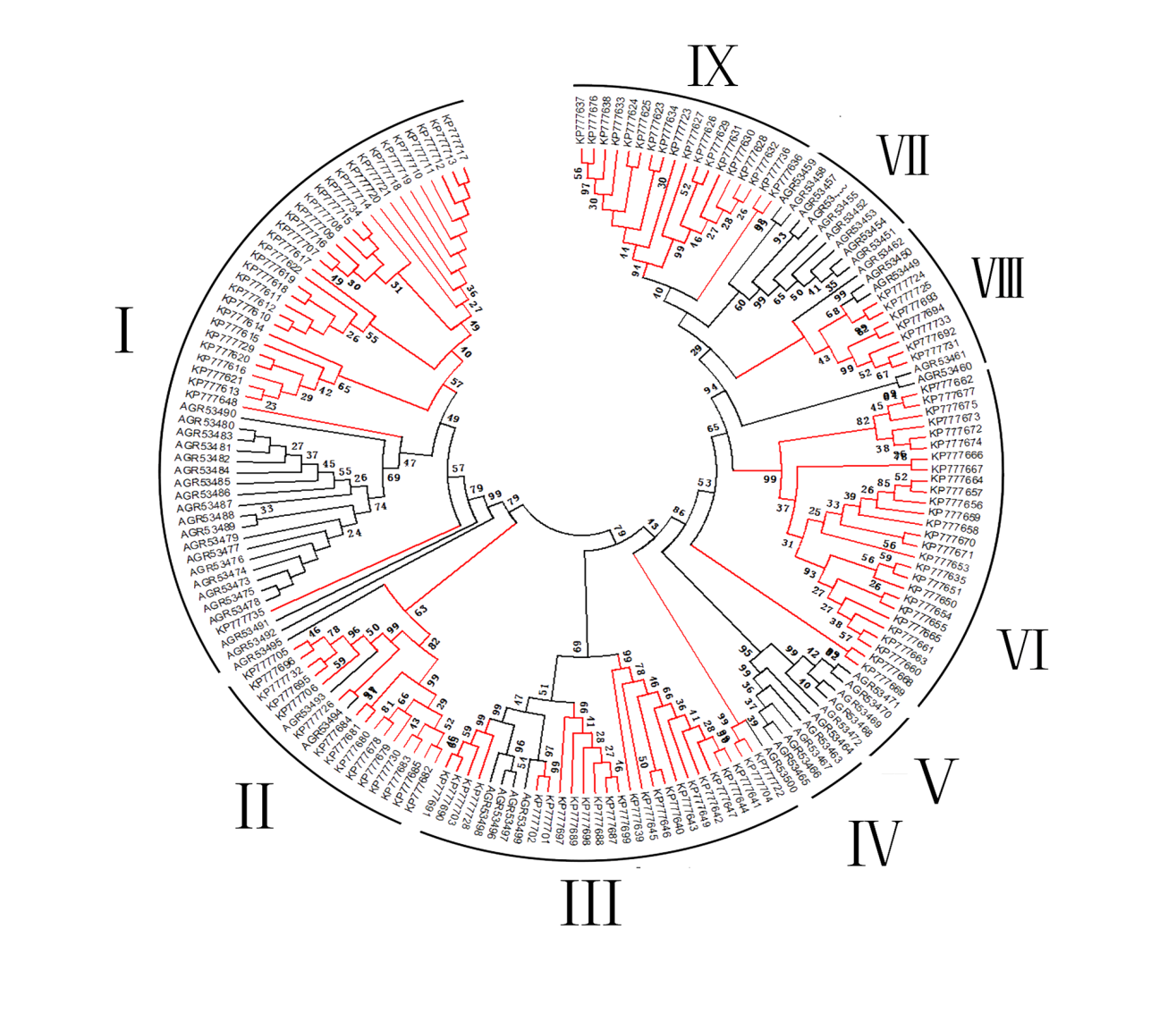


**Figure S3. Phylogenetic tree of putative toxin precursors from *D. sulfurous* and *D.mizhoanus*.** The phylogenetic analysis was conducted by using the Neighbor-Joining method of the MEGA 5 software package. *D. sulfurous* and *D. mizhoanus* toxin precusors were marked with red and black lines, respectively.
