# Supplementary material for: A Comparative Analysis of the Venom Gland Transcriptomes of the Fishing Spiders Dolomedes mizhoanus and Dolomedes sulfurous
Source: PLoS One. 2015 Oct 7;10(10):e0139908. doi: 10.1371/journal.pone.0139908 (PMC4596850; doi:10.1371/journal.pone.0139908)
Supplement: S1 Table — (DOCX) [file pone.0139908.s004.docx]

| **Table S1. Closest matches between molecular weights (MW) of peptide toxins determined by MALDI-TOF MS and calculated from putative mature toxin sequences of *D.sulfurous*** | | | | | |
| --- | --- | --- | --- | --- | --- |
| **HPLC**  **elution time (min)** | **MW^a^** | **GeneBank accession #** | **MW^b^** | **△MW** | **Mature toxin sequences** |
| 23.64 | 6829.30 | KP777634 | 6828.7 | 0.6 | ACVPRGQSCNRDCDCCAGDWDHCNIWGTCVQGTPRDCLDKQKNCAVKPKKCIISSQNRRNQ |
| 33.68 | 7152.65 | KP777696 | 7150.9 | 1.75 | KYCSRSFDCDEGMCCTGGSFNRHCQGLAEDGRPCQRPNEYDHYSTGCPCQEGLICSIINYCQKA |
| 43.44 | 7716.43 | KP777670 | 7716.9 | 0.47 | SCVSPGQVCKDDCDCCVNNNYCHCPLWGILGANSCSCIFGDDIVCKKRMGKCKRNRPQKCPTSRSTRRR |
| 44.44 | 7243.79 | KP777636 | 7244.2 | 0.41 | ACVHRVQSCNNDCDCCVGDWDTCSCTFGYFCSCVPGPARVCFEEQENCAVKPKKCITTVQNKRNQ |
| 44.44 | 7721.56 | KP777692 | 7722.9 | 1.34 | ACKNTGESCDNDCDCCAGEWNSCSCTLGLFFCSCVSGTMRDCMLKQEKCAVKPKVCKRPGQNPRQHKNRP |
| 44.81 | 8080.38 | KP777618 | 8080.0 | 0.38 | ELYCPKPSDPNCNLGYKINHCCSQAECRTGDVCCVQPCGTVCRRGSNTGGGERFVDGSECQLGQVWRSGWSDIF |

Note: ^a^Molecular weights determined by MALDI-TOF MS; ^b^molecular weights calculated by putative mature toxin sequences
