# Supplementary material for: A Comparative Analysis of the Venom Gland Transcriptomes of the Fishing Spiders Dolomedes mizhoanus and Dolomedes sulfurous
Source: PLoS One. 2015 Oct 7;10(10):e0139908. doi: 10.1371/journal.pone.0139908 (PMC4596850; doi:10.1371/journal.pone.0139908)
Supplement: S2 Table — (DOCX) [file pone.0139908.s005.docx]

| **Table S2. Molecular characters of the representative toxins in the families from the spider *D.sulfurous*.** | | | | | | | | | |
| --- | --- | --- | --- | --- | --- | --- | --- | --- | --- |
| Family | Representative  toxin | Precursor | Signal peptide | Propeptide | Propeptide processing signal | Mature peptide | Cysteine  arrangement | Extra C-terminal  residue | Function or sequence identity |
| A | KP777620 | 95aa | 20 | NO | NO | 75 | c-c-cc-c-cc-c-c-c | G | 59% identity to LSTX-Q4 |
|  | KP777621 | 95aa | 20 | NO | NO | 75 | c-c-cc-c-c-c-c-c | G | 78% identity to DMTX-60 |
|  | KP777729 | 80 aa | 20 | NO | NO | 60 | c-c-cc-c-cc-c-c-c | NO | 41% identity to AgorTX_A5 |
| B | KP777722 | 72aa | 25 | 12 | PEER | 35 | c-c-cc-cxc-cxc | NO | Insecticidal neurotoxin |
| C | KP777637 | 113aa | 16 | 35 | VEAR | 62 | c-c-c-cc-c-c-c-c-c | G | 70% identity toDMTX-207 |
|  | KP777723 | 104aa | 16 | 35 | EEAR | 53 | c-c-c-cc-c-c-c-c | NO | 73% identity to DMTX-154 |
| D | KP777641 | 90aa | 22 | 17 | EEPR | 51 | c-c-cc-cxc-cxc | GK | 41% identity to PNTx3-2, Ca^2+^ channels modulator |
|  | KP777728 | 83aa | 22 | 12 | ELAR | 49 | c-c-cc-cxc-cxc | GR | 47% identity to PNTx3-2,Ca^2+^ channels inhibitor |
|  | KP777701 | 81aa | 22 | 16 | DEER | 43 | c-c-cc-cxc-cxc | GK | 48% identity to PNTx3-2, Ca^2+^ channels modulator |
| E | KP777664 | 124aa | 16 | 38 | EEAR | 70 | c-c-cc-cxc-cxc-c-c-c | G | 48% identity to LSTX-L10 |
| F | KP777668 | 113aa | 21 | 17 | EEER | 75 | c-c-c-cc-cxc-cxc-c-c-c | GR | 39% identity to DMTX-174 |
|  | KP777692 | 121aa | 16 | 35 | EEER | 70 | c-c-c-cc-cxc-cxc-c-c-c | NO | 62% identity to DMTX-104 |
| G | KP777696 | 83aa | 19 | NO | NO | 64 | c-c-cc-c-c-cxc-c-c | NO | 51% identity to HNTX-XIV |
| H | KP777681 | 105aa | 19 | 30 | CEKR | 56 | c-c-cc-c-c-cxc-c-c | NO | 89% identity to CSTX-20 |
| The UniProtKB/Swiss-Prot accession numbers for the LSTX-Q4, AgorTX_A5, PNTx3-2, LSTX-L10, HNTX-XIV, CSTX-20 are B6DCY1, Q5Y4V9, O76201, B6DD28, D2Y2C0, B3EWT5.The GenBank accession numbers for are DMTX-60, DMTX-154, DMTX-207, DMTX-174, DMTX-104, are AGR53488, AGR53458, AGR53459, AGR53450, AGR53449. | | | | | | | | | |
